# Pulsed Electromagnetic Field Stimulation of Bone Healing and Joint Preservation: Cellular Mechanisms of Skeletal Response

**DOI:** 10.5435/JAAOSGlobal-D-19-00155

**Published:** 2020-05-18

**Authors:** Ruggero Cadossi, Leo Massari, Jennifer Racine-Avila, Roy K. Aaron

**Affiliations:** From the IGEA, Clinical Biophysics, Carpi, Italy (Dr. Cadossi), the Department of Biomedical and Specialty Surgical Sciences, Azienda Ospedaliero-Universitaria di Ferrara, Arcispedale Sant'Anna, University of Ferrara, Ferrara, Italy (Dr. Massari), and the Department of Orthopaedics, Warren Alpert Medical School, Brown University, Providence, RI (Ms. Racine-Avila and Dr. Aaron).

## Abstract

The US FDA has approved pulsed electromagnetic fields (PEMFs) as a safe and effective treatment for nonunions of bone. Despite its clinical use, the mechanisms of action of electromagnetic stimulation of the skeleton have been elusive. Recently, cell membrane receptors have been identified as the site of action of PEMF and provide a mechanistic rationale for clinical use. This review highlights key processes in cell responses to PEMF as follows: (1) signal transduction through A_2A_ and A_3_ adenosine cell membrane receptors and (2) dose-response effects on the synthesis of structural and signaling extracellular matrix (ECM) components. Through these actions, PEMF can increase the structural integrity of bone and cartilage ECM, enhancing repair, and alter the homeostatic balance of signaling cytokines, producing anti-inflammatory effects. PEMFs exert a proanabolic effect on the bone and cartilage matrix and a chondroprotective effect counteracting the catabolic effects of inflammation in the joint environment. Understanding of PEMF membrane targets, and of the specific intracellular pathways involved, culminating in the synthesis of ECM proteins and reduction in inflammatory cytokines, should enhance confidence in the clinical use of PEMF and the identification of clinical conditions likely to be affected by PEMF exposure.

The musculoskeletal system is highly responsive to its physicochemical environment. Bone and cartilage cells respond to changes in mechanical stress, fluid flow, pH, and pO_2_ by altering their phenotype and expressing a range of signaling and structural molecules that result, in particular, in an altered extracellular matrix (ECM) organization and associated biomechanical properties. Response to mechanical stress is perhaps the best recognized and intuitively obvious of skeletal environmental conditions, facilitating adaptation and modeling to changing biomechanical and environmental requirements perhaps through intermediary strain-associated signaling events. In addition to mechanical stress, skeletal tissues, both bone and cartilage, demonstrate an exquisite sensitivity to electrical and electromagnetic stimulation.

Responses of skeletal cells to pulsed electromagnetic field (PEMF) have been exploited therapeutically with devices that expose tissues to appropriately configured fields to stimulate ECM synthesis for bone and cartilage repair. This review highlights key processes in cell responses to PEMF as follows: (1) signal transduction through cell membrane adenosine receptors (ARs), (2) the activation of osteoinductive pathways, and (3) the synthesis of skeletal ECM including structural and signaling molecules. These actions are reflected physiologically in bone as the healing of fractures, osteotomies, and nonunions, and, in joints, as the modulation of cartilage damage and reduction in catabolic and inflammatory cytokines in arthritis. Understanding the cellular responses to PEMF will inform clinical studies, may point to key issues that need further investigation, and will be relevant in promoting bone and cartilage repair, tissue engineering and regeneration in a repair mode, and damping inflammation in arthritis. Understanding the pathways of the activity of PEMFs provides a solid mechanistic basis for their clinical use.

## Mechanisms of Cellular Responses to Pulsed Electromagnetic Field Exposure

### Signal Characterization and Dosimetry

In the past 20 years, the approach to the study of the biological effects of PEMF has changed notably and the investigation methodology of pharmacology has been adopted through (1) description of the relevant physical parameters: frequency, amplitude, pulse shape, and duration; (2) dose-response effects; and (3) investigation of the mechanisms at the molecular level. The relationships among PEMF signal characteristics, exposure conditions, dosimetry, and biological responses have been investigated. For proteoglycan synthesis in bovine cartilage explants, the largest effect has been observed with 1.5 mT magnetic field peak value and 4 hours of stimulation.^1^ Recently, Parate et al^2^ have extensively tested PEMF effects on the chondrogenic differentiation of human mesenchymal stem cells (MSCs) exposed to various magnetic field amplitudes and stimulation times. They reported a dose-dependent increase in MSC chondrogenesis up to an amplitude of 2 mT and a duration of 10 minutes of exposure, showing then decreased chondrogenic activity for higher doses or longer exposure times, indicating a dosing window of amplitude and exposure duration. In a study examining the effects of power frequency (60 Hz) fields on a model of endochondral ossification (EO), an amplitude dose effect was observed on chondrogenesis measured with biochemical, immunohistochemistry, and molecular end points with a clear maximum response at 0.1 mT.^3^ A maximum response in terms of daily exposure time was at 7 to 9 h/d with lesser response seen at shorter and longer exposure times.

### Membrane Responses to Pulsed Electromagnetic Field Through Adenosine Membrane Receptors

There is strong evidence to support a role for adenosine and its receptors in bone homeostasis and in skeletal pathology, including osteoporosis and arthritis.^4^ Furthermore, adenosine, acting through the A_2A_ receptor, inhibits osteoclast differentiation and increases the rate of new bone formation in bone defects.^5^ A_2A_ signaling also promotes the Wnt/β-catenin pathway regulating bone formation.^6^

**Figure 1:**
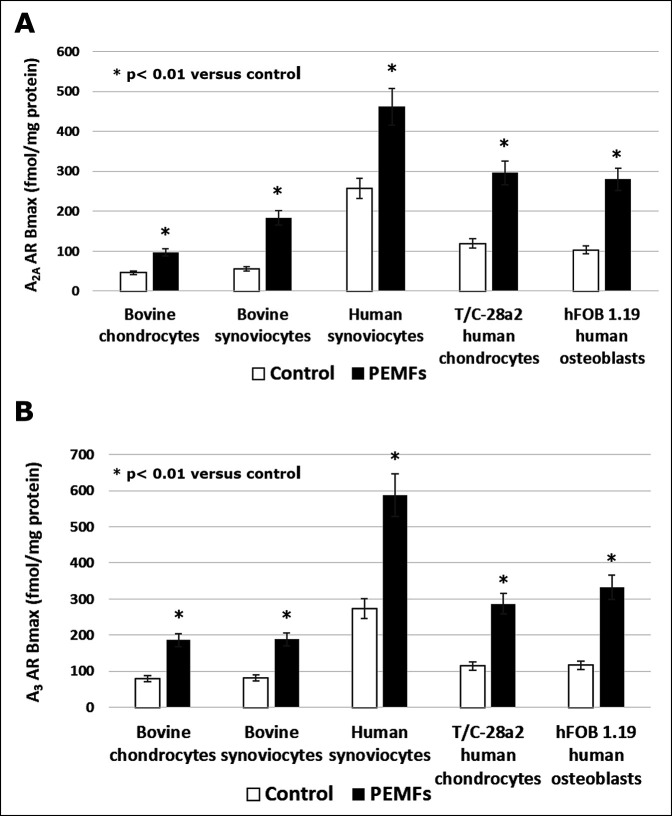
Bar graph showing A_2A_ AR (**A**) and A_3_ AR (**B**) density in bovine chondrocytes and synoviocytes, human synoviocytes, T/C-28a2 human chondrocytes, and hFOB 1.19 human osteoblasts in the absence and in the presence of PEMFs. AR = adenosine receptor, PEMF = pulsed electromagnetic field. (Reproduced with permission from Varani K, Vincenzi F, Ravani A, et al: Adenosine receptors as a biological pathway for the anti-inflammatory and beneficial effects of low frequency low energy pulsed electromagnetic fields. *Mediators Inflamm* 2017;2017:2740963.)

Although the transmembrane signal recognition processes of PEMF are incompletely understood, the specific mechanism of interaction between PEMF and the cell membrane was reported by Varani et al.^7^ They identified for the first time that ARs were the main target of PEMF stimulation in inflammatory cells; ARs play a pivotal role in the regulation of inflammatory processes, with both proinflammatory and anti-inflammatory effects.^8^ It has been demonstrated that PEMF exposure induces a notable increase in A_2A_ and A_3_ AR density on the cell membrane of chondrocytes, synoviocytes, and osteoblasts^8^ (Figure 1). Notably, A_1_ and A_2B_ receptors were not influenced by the same exposure conditions. Moreover, in the presence of the specific A_2A_ receptor agonist, PEMF exposure was able to synergize with the agonist and induce a notable increase in intracellular cyclic adenosine monophosphate (cAMP) levels. On the contrary, the presence of the specific A_2A_ receptor antagonist blocked the effects of both the agonist and PEMF stimulation, suggesting that PEMFs specifically act through the activation of A_2A_ ARs with a pharmacologic-like mechanism. The agonist activity of PEMF for the A_2A_ and the A_3_ ARs is particularly relevant because it inhibits the NF-kB pathway, which is a key regulator of the expression of matrix metalloproteinases and of several genes involved in responses to inflammation.^9^ Cohen et al^10^ showed in vivo that an experimental A_2A_ agonist drug reduced cartilage damage in a rabbit model of septic arthritis of the knee. These observations formed the basis for the application of PEMF for chondroprotection of articular cartilage from the catabolic effects of joint inflammation, as discussed in more detail later.

### Activation of Osteoinductive and Angiogenic Pathways by Pulsed Electromagnetic Field

The stimulation of growth factors and cytokines by PEMF as intermediary activation mechanisms has been summarized by Aaron et al.^11^ They presented the results of 9 studies showing activation by PEMF of the transforming growth factor (TGF)-β gene family, including bone morphogenetic proteins (BMPs) 2 and 4, and increased synthesis of corresponding proteins in a variety of skeletal models. In a detailed study of the effects of PEMF on TGF-β in a model of EO, it was shown that PEMF exposure increased chondrogenic differentiation and EO in association with a 343% increase in TGF-β immunopositive cells.^12^ PEMF exposure was observed to enhance, but not to disorganize, the developmental processes of chondrogenic differentiation and EO. Since those publications, several studies have appeared showing that PEMF exposure stimulates the Wnt/β-catenin signaling pathway resulting in osteogenesis. One study demonstrated improved trabecular microarchitecture by PEMF through activation of the Wnt/β-catenin pathway.^13^ Osteogenic differentiation of osteoblastic cells is increased by PEMF through the Wnt signaling pathway.^14^ PEMF has been shown to upregulate the canonical Wnt ligands, Wnt1,3a, and 10b, in association with increases in bone mass and strength.^15^ PEMF stimulation has also been shown to affect the Wnt signaling pathway in MC3T3-E1 osteoblast-like cells. Zhai et al^16^ demonstrated that PEMF exposure markedly enhanced the expression of components of the Wnt canonical signaling pathway, such as Wnt1, LRP6, and β-catenin. In ovariectomized rats, Jing et al^17^ showed that PEMF modulates bone microarchitecture and strength through the activation of the Wnt/LRP5/β-catenin signaling pathway. Recently, Wu et al^18^ proposed an interdependent Wnt/Ca2+ and Wnt/β-catenin signaling pathway for PEMF-induced osteoblastogenesis in C3H10T1/2 mesenchymal cells, suggesting that both Wnt canonical and noncanonical pathways are involved in osteogenic differentiation.

Angiogenesis is an important component of new bone formation including fracture healing and is deficient in nonunions. PEMF has been shown to increase angiogenesis and perfusion in a number of skeletal-related models.^19-21^ The mechanism of stimulation of angiogenesis by PEMF appears to be dependent on the stimulation of fibroblast growth factor 2, but not vascular endothelial growth factor. In femurs harvested from PEMF-treated mice, Goto et al^22^ reported increased expression levels of angiopoietin-2 and fibroblast growth factor 2 compared with control mice.

A variety of other pathways of intracellular and extracellular PEMF signaling have been described, perhaps dependent on the model system used. Petecchia et al^23^ showed that PEMFs enhance the early stages of osteogenesis in bone marrow stem cells (BMSCs) by increasing the expression of functional L-type voltage-gated calcium channels and the concentration of cytosolic-free calcium. Moreover, recent data from Bagheri et al^24^ showed that PEMF stimulates osteogenic differentiation of BMSCs and increases the expression of Notch4, Dll4, Hey1, Hes1, and Hes5 in osteogenic medium compared with controls, indicating that the activation of the Notch pathway is required for PEMF‐stimulated osteogenic differentiation. Poh et al^25^ demonstrated that PEMF induced the activation of protein kinase B (Akt) and the MAPK/ERK signaling cascade and notably upregulated collagen I, alkaline phosphatase, and osteocalcin.

### Pulsed Electromagnetic Field Promotes the Synthesis of Extracellular Matrix in Skeletal Models

**Figure 2:**
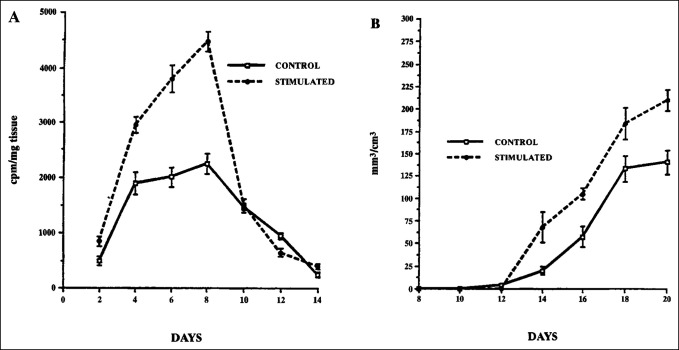
Graph showing the incorporation of radiolabeled sulfate into proteoglycan and bone formation. **A**, Ossicles exposed to PEMF stimulation exhibit a notable increase in proteoglycan synthesis by day 4 of development, peaking during maximal chondrogenesis on day 8 (*P* = 0.001) and falling to control levels coincident with the onset of calcification. **B**, Trabecular bone formation is increased with PEMF stimulation. PEMF = pulsed electromagnetic field. (Adapted from Aaron RK, Ciombor DM: Acceleration of experimental endochondral ossification by biophysical stimulation of the progenitor cell pool. *J Orthop Res* 1996;14:582-589.)

**Figure 3:**
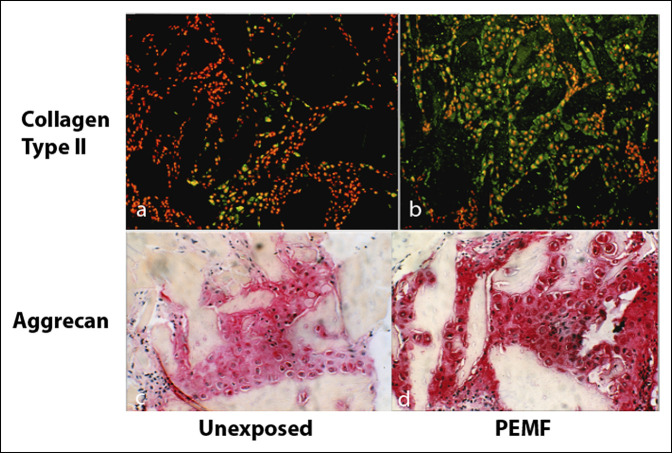
Immunohistochemistry of type II collagen and aggrecan in the DBM-EO model with and without exposure to PEMF. Dark areas in (**A** and **B**) and light areas in (**C** and **D**) are DBM particles. PEMF stimulation increases both ECM molecules. DBM = demineralized bone matrix, ECM = extracellular matrix, EO = endochondral ossification, PEMF = pulsed electromagnetic field

The physiologic mechanism of the response of skeletal cells to PEMF is the synthesis of ECM structural and signaling molecules in the context of repair. In a well-characterized in vivo model of EO, PEMF stimulation has been shown to increase the synthesis of cartilage and bone matrix growth factors and to enhance the proliferation and differentiation of osteoblast-like primary cells.^11^ The demineralized bone matrix–induced EO model developed by Hari Reddi has been extremely useful for examining the details of chondrogenesis and endochondral bone formation, including cell differentiation and ECM synthesis. It has been used in several studies to examine the response to PEMF of mesenchymal cells undergoing EO. In this model, demineralized bone matrix is prepared from rat long bones and is implanted subcutaneously along the thoracic musculature. It induces mesenchymal cell infiltration and differentiation resulting in mature bone, or ossicle, through the process of EO. Chondrogenesis peaks at day 8 of ossicle development, and then, the cartilage matrix is eroded by osteoclasts and replaced with bone that forms into mature trabeculae on days 18 to 20 of development. Exposure to PEMF of animals bearing developing ossicles resulted in an increase in chondrogenesis, on-schedule removal of cartilage, and accelerated bone formation (Figure 2).^26^ Immunohistochemistry demonstrates the spatial increase in proteoglycan induced by PEMF exposure (Figure 3). mRNA for aggrecan and type II collagen, aggrecan synthesis, glycosaminoglycan content, the spatial area of cartilage ECM, and the number of chondrocytes are all increased by PEMF exposure (Table 1). The ratio of chondrocytes to ECM is unchanged, indicating normal morphology with PEMF exposure. In a detailed study of chondrogenesis with this model, proteoglycan and glycosaminoglycan molecular sizes and chemical composition were normal with PEMF stimulation.^27^ The increased expression of cartilage macromolecules, greater immunoreactivity of 3B3 and 5D4 epitopes, and no change in DNA content or 3H-thymidine incorporation suggested increased cell differentiation as the most likely mechanism of PEMF activity. Studies with this model have shown an increase in TGF-β mRNA and protein levels in association with chondrogenic differentiation with rapid progression to EO and bone formation.^12^ The locus of the stimulation of endochondral bone formation by PEMF was shown to reside in the early mesenchymal stage before chondrogenesis.^28^

**Table 1:** Increase in Indices of Chondrogenesis on Day 8 of Ossicle Development by Exposure to PEMF^12^

	Control	PEMF	Percent	*P*
mRNA aggrecan	6.1	22.5	269	0.02
mRNA type II collagen	11.8	21.9	86	0.05
^35^SO_4_ incorporation (cpm/mg)	2166 ± 387	4448 ± 293	105	0.005
GAG content (μg/mg)	1.4 ± 0.2	2.5 ± 0.2	79	0.01
Cartilage area (mm^2^)	24 ± 2.1	148 ± 11.7	517	0.001
Chondrocytes (n)	701 ± 227	3582 ± 675	411	0.005
Chondrocyte/cartilage	29.2	24.2		n.s.

GAG = glycosaminoglycan, PEMF = pulsed electromagnetic field

Wang et al^29^ observed that PEMFs stimulate osteogenic differentiation and mineralization through the activation of the sAC-cAMP-PKA-CREB signaling pathway. Ongaro et al reported that PEMF stimulation increases alkaline phosphatase activity, osteocalcin, and matrix mineralization in MSCs isolated from both BMSCs and adipose tissue–derived MSCs.^9^ In particular, in BMSCs, PEMF showed a synergistic action with BMP-2, an essential growth factor for bone cells.^30^ Moreover, PEMFs have been shown to induce BMP-2 mRNA expression in human bone marrow stromal cells.^31^ Ehnert et al^32^ demonstrated increased proliferation and osteogenic differentiation with PEMF exposure in a coculture system of adipose tissue–derived MSCs and osteoblasts. The result of the signaling processes is to instruct skeletal cells to synthesize structural ECM and signaling molecules, enhance the ability of skeletal tissues to respond to changing physicochemical environments and biomechanical demands, and facilitate repair.

Other models have demonstrated PEMF effects on bone repair. Canè et al,^33^ in holes drilled into the metacarpal bone of male horses, showed that new trabecular growth was 3.4 µm/d in bone exposed to PEMF compared with 1.8 µm/d in controls. In a rat fibular osteotomy model, Midura et al^34^ observed, 9 days after surgery, a 2-fold faster rate of hard callus formation in PEMF-treated limbs, yielding a 2-fold increase in callus volume by 13 to 20 days after surgery. Fassina et al,^35^ using PEMF-stimulated SAOS-2 human osteoblasts, showed enhanced cellular proliferation and increased expression of decorin, fibronectin, osteocalcin, osteopontin, TGF-β1, type I collagen, and type III collagen. Together, the studies represent the rationale for the clinical use of PEMF in promoting bone healing.

## Pulsed Electromagnetic Field Stimulation of Bone Fracture Healing

### Fresh Fractures and Surgical Osteotomies

Clinical PEMF stimulation is widely applied in both the United States and Europe as a noninvasive and safe therapy to promote bone repair. Considerable level 1 evidence has accumulated, demonstrating clinical efficacy of PEMF exposure in accelerating healing of fresh fractures and osteotomies (Table 2). Borsalino, in patients undergoing femoral osteotomy, and Mammi, in patients undergoing tibial osteotomy, demonstrated the efficacy of PEMF in promoting bone healing.^36,37^ Fontanesi, in fresh tibial fractures, and Faldini, in femoral neck fractures, reported, respectively, a reduction in time to union and an increase in the percentage of fracture healing in PEMF-stimulated patients compared with controls.^38,39^

**Table 2:** Level I Studies of Bone Healing With PEMF^9^

Reference	Clinical application
Poli et al, 1985	Congenital nonunion, intramedullary nail fixation
Borsalino et al^36^	Femoral intertrochanteric osteotomies
Sharrard, 1990	Tibial nonunion, cast
Mammi et al^37^	Tibial osteotomies
Capanna et al, 1994	Osteotomy, tumor resection, and bone graft
Simonis et al, 2003	Tibial nonunion, osteotomy, and external fixator
Faldini et al^39^	Femoral neck fractures treated with screws
Hannemann et al, 2012	Acute scaphoid fractures treated with cast
Shi et al^45^	Nonunion of long bone, nail, and plate

PEMF = pulsed electromagnetic field

### Fracture Nonunions

**Figure 4:**
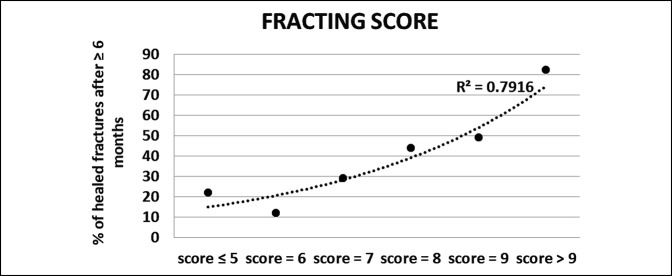
Graph showing that the FRACTING score reliably identifies fractures healing in greater than 6 months (delayed union). Multiple risk factors are combined to produce the FRACTING score.

In the United States, PEMF exposure is FDA-approved for the treatment of fracture nonunions. Considerable clinical evidence supports the use of PEMF for nonunions with reported healing rates for nonunions after PEMF stimulation between 73% and 85%.^40^ Hinsenkamp et al^41^ reported a success rate above 70% in a European multicenter study including 308 patients. Traina et al^42^ achieved higher success rates (87.8% versus 69%) and shorter healing time with PEMF stimulation compared with surgery in nonunions of at least 9 months from trauma.^42^ Notably, in the PEMF group, the presence of infection did not negatively affect the effectiveness of the stimulation.^42^ In a Spanish retrospective cohort study of tibial nonunions, Cebriàn et al^43^ reported a healing rate of 91% in the PEMF-stimulated group compared with 83% of the control group. Recently, in a study of 1,382 patients with fracture nonunions, Murray and Pethica^44^ showed that patients who used PEMF stimulation for 9 hours or more per day healed 76 days earlier than patients who used PEMF stimulation for 3 hours or less per day. In a prospective randomized controlled study, Shi et al^45^ reported that early application of PEMF notably increased union rates in patients with long-bone delayed unions. Cost-benefit analysis appeared to justify early PEMF application for fractures that are likely to require a long time to heal. In this regard, it is worth highlighting the effort undertaken by the Italian group of orthopaedic surgeons, who developed the “FRACTING score” that can be applied to reliably estimate months needed for fracture healing and to accurately identify fractures at risk of nonunion^46^ (Figure 4).

## Pulsed Electromagnetic Field Effects on Cartilage and Joint Biology

### Articular Cartilage

Because cartilage is composed of a highly electrically charged ionic gel matrix, mechanical strain produces electrokinetic events, including streaming potentials, in addition to other physicochemical changes that may act as cell signaling events. Cartilage explants in vitro, and cartilage models in vivo, have been quite responsive to externally applied PEMFs with the response expressed as enhanced synthesis of ECM and supporting cytokines, particularly of the TGF-β/BMP family. Synthesis of cartilage ECM molecules with electromagnetic stimulation has been summarized in several reviews.^47-49^

**Figure 5:**
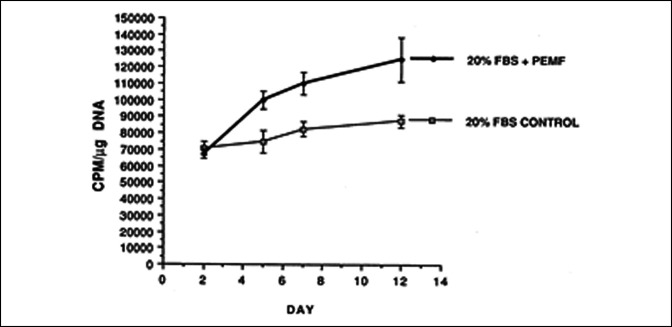
Graph showing proteoglycan synthesis in adult bovine articular cartilage aged 4 to 6 years. Exposure to PEMF notably increases macromolecular radiolabeled sulfate into proteoglycans during 14 days of culture.^50^ FBS = fetal bovine serum, PEMF = pulsed electromagnetic field

The responsiveness of bovine cartilage explants to PEMF exposure has been studied in some detail. Using macromolecular radiolabeled sulfate incorporation into proteoglycan, notable increases in proteoglycan synthesis have been observed in PEMF-exposed, compared with unstimulated control, bovine cartilage explants (Figure 5).^50^ The proteoglycan molecules in PEMF-exposed explants were of equivalent size and degree of sulfation as were the controls, indicating the synthesis of normal proteoglycan molecules. An interesting observation was that exposure to PEMF increased proteoglycan synthesis in older cartilage to the level of that of cartilage from younger animals. PEMF stimulation of proteoglycan synthesis has been shown to be additive to that of IGF-1.^51^ PEMF + IGF-1 increased proteoglycan synthesis by 56% at maximum dosage compared with 25% by IGF-1 alone in bovine cartilage explants. Also, in bovine articular cartilage explants, PEMF stimulation has been shown to promote proteoglycan synthesis in the presence of IL-1β, thus antagonizing the catabolic activity of proinflammatory cytokines.^52^

In human osteoarthritic cartilage explants derived from knee arthroplasty, PEMF exposure stimulated proteoglycan synthesis in less pathologically involved cartilage, as shown by Mankin histological-histochemical scores.^53^ In cartilage with Mankin scores of eight or greater (of a possible score of 14), PEMF had no effect, suggesting that therapeutic effects of the fields were best expected in early osteoarthritis (OA), before major structural matrix damage. The increase in proteoglycan synthesis was paralleled by an increase in stiffness, or aggregate modulus, exhibiting a dose effect of exposure duration: 1-hr exposure was more effective than 4-hour exposure in increasing cartilage stiffness.

### Articular Inflammation and Cytokines

The detrimental effect of inflammation on joint cartilage results in cartilage degradation and arthritis. An agonist activity for the A_2A_ receptor can physiologically counteract inflammation and inhibit synthesis and release of proinflammatory cytokines. PEMF has a strong agonist activity for ARs. PEMF exposure induces a specific overexpression of A_2A_ and A_3_ ARs in human chondrocytes. In particular, PEMFs have been shown to potentiate the responses of A_2A_ and A_3_ AR agonists on cAMP production, suggesting a synergistic effect of biophysical stimulation and A_2A_ and A_3_ AR activation. PEMFs also synergized with A_2A_ AR agonists in potentiating the proliferative action on both human chondrocytes and osteoblasts.^8^ PEMF stimulation, through the activation of A_2A_ and A_3_ ARs, has been shown to inhibit the NF-kB pathway, leading to decreased release of TNF-α and IL-1β by human synoviocytes and chondrocytes and reduced synthesis of prostaglandin E2 (PGE2) and COX-2 by bovine synoviocytes.^9^ Ongaro et al demonstrated in human synovial fibroblasts from patients with OA that PEMF stimulation reduces the synthesis of inflammatory mediators such as PGE2, IL-6, and IL-8, while stimulating the release of the anti-inflammatory interleukin-10.^9^ These observations suggest that PEMFs exert a proanabolic effect on the cartilage matrix and a chondroprotective effect counteracting the catabolic effects of inflammation in the joint environment. Together, these data suggest that PEMF may prevent or limit articular cartilage degradation, leading to joint preservation.

### In Vivo Models of Osteoarthritis

**Figure 6:**
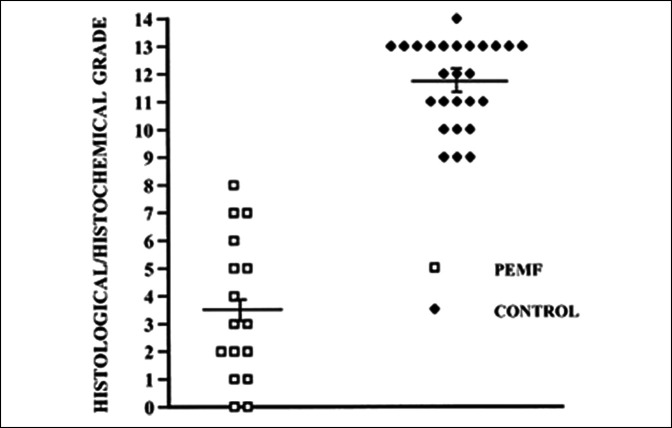
Graph showing the histological/histochemical grade of tibial articular cartilage. Mean grade of control tibias was 11.7 ± 0.3 compared with 3.5 ± 0.7 of PEMF-treated cartilage (*P* = 0.0001), reflecting the preservation of cartilage morphology in the PEMF-treated group. PEMF = pulsed electromagnetic field. (Reprinted with permission from Ciombor DM, Aaron RK, Wang S, Simon B: Modification of osteoarthritis by pulsed electromagnetic field-a morphological study. *Osteoarthritis Cartilage* 2003; 11:455-462.)

The chondroprotective hypothesis has been tested in vivo in the Dunkin-Hartley guinea pig, an animal model that develops spontaneous OA at 9 to 12 months of age. The guinea pig exhibits the stereotypical loss of articular cartilage Safranin-O staining, surface fibrillation, and deep matrix clefts as observed in human OA. Preservation of ECM was observed in guinea pigs exposed to PEMF, and Mankin histological-histochemical scores demonstrated substantial differences in matrix and cell loss between the two groups (Figure 6).^54,55^ The pattern of articular cytokines was quite different between the PEMF-exposed and control groups. PEMF exposure resulted in an increase in TGF-β immunopositive cells and a corresponding decrease in IL-1 and matrix-degrading enzymes, suggesting that the cytokine environment of the arthritic joints was modified in this model (Table 3). Parallel studies with the same animal model concentrated on structural preservation of bone and cartilage by PEMF exposure.^55^ This study demonstrated both lower Mankin scores, indicating the preservation of cartilage ECM, and reductions in subchondral bone thickness in PEMF-treated animals compared with untreated controls, indicating reductions in pathological bone remodeling (Table 4).

**Table 3:** Immunopositive Cells (Per Unit Area) With PEMF Exposure^54^

	Control	PEMF	% Change	*P*
MMP-13	7.2 ± 0.8	0.0 ± 0.0	—	0.01
MMP-3	13.8 ± 1.9	8.4 ± 1.0	−39	0.02
IL-1	13.9 ± 2.3	7.2 ± 0.6	−48	0.01
IRAP	10.5 ± 1.0	17.3 ± 1.1	+65	0.003
TGF-β	20.0 ± 3.5	34.4 ± 2.8	+72	0.006

IRAP = Interleukin-1 receptor antagonist protein, MMP = matrix metalloproteinase, PEMF = pulsed electromagnetic field, TGF-β = transforming growth factor β

**Table 4:** Cartilage Histological Score and Subchondral Bone Thickness Results for Sham-Treated and PEMF-Treated Animals

Measurement Site	Cartilage Histological Score		Subchondral Bone Thickness (μ)	
	Sham Treated	PEMF Treated	Sham Treated	PEMF Treated
Medial tibia plateau	10.9 ± 0.9	3.9 ± 0.7^a^	284.0 ± 38.0	242.2 ± 27.3^a^
Medial femoral condyle	7.4 ± 0.8	2.5 ± 0.7^a^	325.0 ± 32.8	271.0 ± 27.5^a^
Lateral tibia plateau	6.4 ± 1.0	1.6 ± 0.4^a^	304.3 ± 31.4	265.4 ± 29.2^a^
Lateral femoral condyle	5.9 ± 0.9	1.8 ± 0.5^a^	306.5 ± 34.4	261.7 ± 28.3^a^

PEMF = pulsed electromagnetic field

Data adopted from Fini M, Giavaresi G, Torricelli P, Cavani F, Setti S, Cane V, Giardino R: Pulsed electromagnetic fields reduce knee osteoarthritic lesion progression in the aged Dunkin Hartley guinea pig. *J Orthop Res* 2005;23:899–908.

a *P* < 0.05.

In agreement with these findings, Yang et al^56^ reported that preemptive and early PEMF treatment in low-dose monosodium iodoacetate–treated rats increased bone and cartilage synthesis, while decreasing bone and cartilage degradation. They concluded that the efficacy of PEMF exposure on OA is associated with an early application of treatment. Additional important PEMF effects on cytokine synthesis were observed in adult sheep treated with autologous osteochondral grafts. PEMF favored graft integration and prevented graft reabsorption. Notably, lower levels of inflammatory catabolic cytokines (IL-1β and TNF-α) and higher concentration of TGF-β were measured in the synovial fluid of PEMF-treated animals compared with controls. On the basis of these studies collectively, Fini et al^55^ suggested that PEMF stimulation could play a significant role in tissue engineering treatment protocols.

### Clinical Observations of Pulsed Electromagnetic Field Effects After Joint Surgery

Joint surgery modifies the local cytokine environment resulting in increased levels of proinflammatory cytokines in synovial fluid that can have a catabolic effect on cartilage ECM, ultimately leading to the development of OA.^57^ Based on its expected chondroprotective and anti-inflammatory effects, PEMF treatment has been applied to the knee joint, after surgery, to preserve cartilage integrity and to promote functional recovery.

After arthroscopic treatment of cartilage lesions in the knee, Zorzi et al^58^ reported a notable reduction in the percentage of patients using NSAIDs in the PEMF-treated group compared with the controls. They also reported improvement in functional recovery in PEMF-treated patients 90 days after surgery and the maintenance of such advantage up to 3 years of follow-up. PEMF stimulation has also been shown to be effective in patients undergoing anterior cruciate ligament reconstruction. In a randomized double-blind study, Benazzo et al^59^ showed reduction in the recovery time and early return-to-sport activity. Similar results have been reported by Osti et al^60^ in patients undergoing microfracture for the treatment of OA of the knee. PEMF stimulation was able to markedly improve the American Orthopaedic Foot and Ankle Society score and to markedly reduce pain levels in patients with talar osteochondral lesions treated with collagen scaffolds seeded with bone marrow–derived cells at 6 and 12 months of follow-up.^61^ Similar results have been obtained with PEMF stimulation in association with matrix-assisted autologous chondrocyte implantation in the treatment of chondral lesions of the knee.^62^ Finally, two recent Italian studies on patients undergoing total knee arthroplasty reported notably reduced pain and knee swelling, together with improved functional scores in PEMF-exposed patients.^63,64^

As a conservative treatment, PEMF stimulation has been used to relieve pain in patients with early-stage OA. Thamsborg et al indicated that, compared with older subjects, younger patients (<65 years) had a better functional outcomes.^65^ Furthermore, in early-stage OA, Gobbi et al^66^ reported notable improvement in pain, knee function, and quality of life at 1-year follow-up in the PEMF-stimulated patients. Similar results have been described by Servodio Iammarrone et al^67^ in patients with patellofemoral pain; PEMF stimulation was able to improve joint function, favor pain resolution, and shorten the time to return-to-sport activity.

## Summary

In 1979, the US FDA approved PEMF as a safe and effective treatment for nonunions of bone. Since then, the use of PEMF stimulation for bone repair has grown both in the United States and in Europe. In the United States, a survey showed that 72% of hospitals offer bone repair stimulation treatments for fractures that fail to heal. Analogous to pharmacodynamics as a key step in drug adoption, PEMF dose-response effects provide a solid conceptual basis for clinical use. The local field of biological activity, as opposed to systemic effects, represents a notable advantage of PEMF together with a lack of negative adverse effects in relation to its efficacy.

Despite its clinical use, the mechanisms of action of electromagnetic stimulation of the skeleton have been elusive, and PEMF has been viewed as a “black box.” In the past 25 years, research has been successful in identifying cell membrane receptors and osteoinductive pathways as sites of action of PEMF and provides a mechanistic rationale for clinical use. Understanding of PEMF membrane targets, and of the specific intracellular and extracellular pathways involved, culminating in the synthesis of ECM proteins and reduction in inflammatory cytokines, should enhance confidence in the clinical use of PEMF and the identification of clinical conditions likely to be affected by PEMF exposure.

The biological effects of PEMF treatment and favorable effects on the skeletal system are the result of notable research efforts conducted internationally by the orthopaedic community, and they have attracted much interest from other medical specialties such as wound and tendon healing, rheumatology, and neurology that may be able to take advantage of the experiences developed with bone and cartilage treatments.
